# Evaluating for Differences by Race/Ethnicity in the Association Between Income and Gambling Disorder

**DOI:** 10.1007/s10899-020-09941-6

**Published:** 2020-04-09

**Authors:** Brendan Day, Geoffrey Rosenthal, Fiyinfolu Adetunji, Andrea Monaghan, Christina Scheele, J. Kathleen Tracy

**Affiliations:** 1 Department of Epidemiology and Public Health, University of Maryland School of Medicine, 660 W. Redwood Street, Howard Hall, 102D, Baltimore, MD 21201 USA; 2 Department of Pediatrics, University of Maryland School of Medicine, Baltimore, MD 21201 USA; 3 Department of Medicine, University of Maryland School of Medicine, Baltimore, MD 21201 USA; 4 Maryland Center of Excellence on Problem Gambling, University of Maryland School of Medicine, Columbia, MD 20145 USA

**Keywords:** Income, Race, Ethnicity, Gambling disorder, Risk factor

## Abstract

Multiple studies show an increased prevalence of gambling disorder among African Americans compared to whites. However, few studies take an analytic approach to understanding differences in risk factors by race/ethnicity. Income is inversely associated with gambling disorder; we hypothesized that this association would vary by race/ethnicity. The main objective was to evaluate whether the association between income and gambling disorder varies by race/ethnicity. With data from the baseline visit of a prospective cohort study, Prevention and Etiology of Gambling Addiction Study in the United States, we used multivariable logistic regression analysis to determine whether the association between income and gambling disorder varies by race/ethnicity. 1164 participants were included in the final analyses. Measures included: demographics (age, sex, race/ethnicity, education, employment, annual household income), veteran status, marital status, homelessness, smoking, substance abuse, alcohol abuse, marijuana use, and lifetime gambling disorder diagnosis as derived from Alcohol Use Disorder and Associated Disabilities Interview Schedule. There was no evidence of effect modification by race/ethnicity in the association between income and gambling disorder (global *p* value = 0.17). Income was associated with increased odds of gambling disorder, but only for those with low income (< $15,000; OR 2.27, 95% CI 1.46, 3.53). There was no evidence that the effect of income on gambling disorder varies by race/ethnicity. For all race/ethnicities combined, low income was associated with significantly increased odds of gambling disorder (OR 2.27, 95% CI 1.46, 3.53). Further research is needed to better understand racial/ethnic differences in gambling disorder.

## Introduction

### Background and Rationale

Gambling is common to almost every culture (Okuda et al. 2016) and has been part of human life for thousands of years (Schwartz 2013). Although many people gamble without experiencing negative consequences, for some individuals gambling can develop into a gambling disorder (previously termed pathological gambling), with the potential for devastating consequences on health and well-being (American Psychiatric Association 2013; Petry et al. 2014).

In the United States, gambling disorder affects around 0.4–2% of individuals over their lifetime, with estimates varying depending on the study (Gerstein et al. 1999; Kessler et al. 2008; Petry et al. 2005; Welte et al. 2001). However, gambling disorder does not affect all racial/ethnic groups equally. Numerous studies have shown that gambling disorder is more prevalent among minority groups than whites (Alegría et al. 2009; Barnes et al. 2017; Gerstein et al. 1999; Welte et al. 2001). Although this has been seen for several minority groups in the United States, African Americans represent the largest minority group with gambling disorder prevalence significantly higher than whites (Alegría et al. 2009; United States Census Bureau 2016; Welte et al. 2001). In a study from a large, nationally representative survey done in 2001–2002, Alegría et al. (2009) found that the lifetime prevalence of gambling disorder [using Diagnostic and Statistical Manual of Mental Disorders, 4th edition (DSM-IV) criteria] in African Americans was more than double that of whites (0.9% vs. 0.4%) with a similar difference seen when using the more broadly defined “problem gambling” (2.2% vs. 1.2%). Furthermore, similar findings have been noted by other studies with different samples and varying definitions of gambling disorder (Gerstein et al. 1999; Shinogle et al. 2011; Welte et al. 2001).

Although racial/ethnic differences in gambling disorder prevalence are well documented and consistent across studies, the underlying reasons for such differences are not well understood (Barnes et al. 2017). Since race/ethnicity is likely a proxy for underlying risk factors for gambling disorder rather than a risk factor itself (Okuda et al. 2016), studies exploring differences in risk factors by race/ethnicity are needed for further understanding.

Several cross-sectional studies suggest that income level has an inverse association with gambling disorder (Alegría et al. 2009; Gerstein et al. 1999; Shinogle et al. 2011). In a nationally representative study done in 1999 by Gerstein et al., those in the lowest income group had the highest prevalence of gambling disorder while those in the highest income group had the lowest prevalence of gambling disorder (Gerstein et al. 1999). More recent studies show a similar trend, with gambling disorder prevalence decreasing as income level increases (Alegría et al. 2009; Shinogle et al. 2011). Finally, regarding differences by race/ethnicity, the study by Alegría et al. (2009) showed that African American participants with “disordered gambling” were significantly more likely to be in the low income group compared to whites with “disordered gambling.” Although these findings are descriptive in nature, they suggest that the relationship between income, race/ethnicity, and gambling disorder bears further investigation.

### Objectives and Hypothesis

The purpose of this study was to determine if the association between income and gambling disorder varies by race/ethnicity using baseline data from a parent study, Prevention and Etiology of Gambling Addiction Study in the United States (PEGASUS). The objectives included first evaluating the association between income level and gambling disorder while controlling for potential confounders, then determining if this association differed by racial/ethnic group. We hypothesized that there would be an inverse association between income and gambling disorder, and that the strength of this association would differ by race/ethnicity, with a stronger association seen in African Americans compared to whites.

## Methods

### Study Design, Setting, and Participants

The present study used baseline data from PEGASUS, a 5-year prospective cohort study which began in 2015. The purpose of PEGASUS is to identify risk factors, protective factors, and biological correlates of gambling behavior. Participants recruited were healthy adults aged 18 years and older residing in Maryland who could read and write in English. Individuals with and without problem gambling behavior were recruited with the intent of oversampling those with problem gambling behavior in a ratio of 2:1 (using the NODS-CLiP 3-item screen) (Toce-Gerstein et al. 2009). The proposed sample size was 1500 and the total accrual achieved was 1346 (89.7%). The majority of participants were recruited via targeted social media advertisement. A small number of participants were also recruited via fliers posted at the University of Maryland Baltimore. At the baseline visit, participants completed a battery of self-administered questionnaires including sociodemographic characteristics, health history, and gambling behaviors, which is the source of data for this analysis. The study was reviewed and approved by the Institutional Review Board for the University of Maryland Baltimore. All participants provided written informed consent to participate.

### Measures

The dependent variable was lifetime diagnosis of gambling disorder. This was defined according to the diagnostic criteria for gambling disorder set forth by the DSM-5. Alcohol Use Disorder and Associated Disabilities Interview Schedule-IV (AUDADIS-IV) was used to assess gambling behavior among participants. Although it does not confer a clinical diagnosis, AUDADIS-IV has been used in multiple gambling studies to approximate the prevalence of clinical diagnoses on a population level (Alegría et al. 2009; Barry et al. 2010; Desai and Potenza 2008; Petry et al. 2005, 2014; Subramaniam et al. 2015). Lastly, although AUDADIS-IV is designed for assessing DSM-IV criteria for pathological gambling, there is a very high concordance (> 99%) between the two diagnostic criteria for those with gambling disorder (Petry et al. 2014). This allowed us to adapt the questionnaire to be in keeping with current terminology and identify those with DSM-5 criteria for gambling disorder.

The main independent variable was self-reported annual household income and was categorized in the following four categories: low income (< $15,000), low-middle income ($15,000–< $25,000), high-middle income ($25,000–< $50,000), and high income ($50,000 +). These categories were selected based on cutoffs used for low income in other studies on gambling disorder, as well as to ensure an adequate distribution of participants in each group for the analysis (Alegría et al. 2009; Shinogle et al. 2011).

The following covariates were considered for inclusion in the final model for this analysis: age (18–24, 25–34, 35–44, 45–54, 55–64, 65 +), sex (male, female), race/ethnicity (non-Hispanic white, non-Hispanic African American, ‘Other’), education level (less than high school graduate, high school graduate, more than high school graduate), employment status (employed, unemployed, non-working), veteran status (yes, no), marital status (single, married, divorced/separated/widowed), homelessness (yes, no), current smoker (yes, no), substance abuse (yes, no), alcohol abuse (yes, no), and marijuana use in the past 30 days (yes, no). Past-year substance abuse was defined using the Drug Abuse Screening Test-20 (DAST-20), a reliable and valid instrument for predicting substance abuse (Yudko et al. 2007). As DAST-20 is generally used, we used a score of six or higher to indicate those who screened positive for substance abuse. Alcohol abuse was defined using the Alcohol Use Disorders Identification Test (AUDIT), a 10-item screening instrument used to identify current hazardous and harmful alcohol consumption (Saunders et al. 1993). AUDIT has also proven to be reliable and valid with the generally accepted cut-off point of eight for identifying a possible alcohol problem (Reinert and Allen 2002). For this analysis, we used an AUDIT score of eight or higher to indicate alcohol abuse. Lastly, we chose to categorize the race/ethnicity groups as such since we anticipated certain race/ethnicity groups would be too small to analyze alone.

### Statistical Analysis

The distribution and frequency of variables both for the total sample and for each race/ethnicity group were evaluated. Confounding assessment was performed. This included a directed acyclic graph (DAG), bivariate associations (between covariates and the main independent variable, then between covariates and the dependent variable), and percent change in estimate. Confounders were defined a priori to be those variables in our final model that were (a) part of the minimally sufficient adjustment set from the DAG and/or (b) covariates which were associated with both the main independent variable and dependent variable (*p* value < 0.05) *and* resulted in a greater than 10% change in estimate when added to the unadjusted model. Effect modification by race/ethnicity (effect modifier of interest) and sex (potential effect modifier based on the literature) was evaluated in the unadjusted model. For the final analysis, the following confounders were added to the unadjusted model to obtain an adjusted estimate of the association between income and gambling disorder: age, sex, education, employment, marital status, current smoking, alcohol abuse, and substance abuse. Effect modification by race/ethnicity was also re-evaluated in the adjusted model.

Bivariate associations were evaluated using Chi squared or Fisher’s exact tests. Change in estimate was assessed by adding each covariate to an unadjusted logistic regression model for the association between income and gambling disorder. Effect modification was assessed by adding interaction terms to both unadjusted and adjusted logistic regression models for the association between income and gambling disorder. Significance of interaction terms was determined using a global *p* value (i.e. joint tests using Wald Chi square). Significance of the association between income and gambling disorder was determined using a *p* value for each level of income.

Sensitivity analyses were performed to ensure consistency of our assessment for effect modification by race/ethnicity, first by altering race/ethnicity categories, then by modifying the time frame for the main dependent variable. For race/ethnicity, analyses were performed using a greater number of race/ethnicity categories: white, African American, American Indian/Alaska Native, Asian, and other (including more than one race). For the main dependent variable, analyses were performed after restricting from lifetime to past-year gambling disorder.

Statistical analysis was performed using SAS software Version 9.4 (SAS Institute Inc., Cary, NC, USA). Statistical significance was inferred for results that achieved a *p* value < 0.05.

## Results

### Study Participants

Of the total 1346 participants in the parent study (PEGASUS), the majority were either African American (46.8%) or white (39.5%). The ‘Other’ race/ethnicity group was comprised of 180 individuals (13.4%) who reported their race/ethnicity as ‘other’ or more than one race (n = 78), Asian (n = 63), Hispanic white (n = 21), Hispanic black (n = 12), and American Indian/Alaska Native (n = 6). 182 participants from the total enrolled were excluded from the analyses due to missing or incomplete data for variables of interest, leaving a sample size of 1164 for the final analysis. The variables with the highest amounts of missingness were substance abuse (8.7%), veteran status (5.4%), and alcohol abuse (4.0%), with the remaining variables of interest each missing less than 2%. Compared to those included in the analysis, excluded participants were more likely to have the following characteristics: older age, non-white race, high school education or less, substance abuse, gambling disorder, and no marijuana use. Sample characteristics varied substantially between the three race/ethnicity groups used in the main analysis (Table 1). Except for marijuana use and homelessness, there were statistically significant differences between the groups for each variable analyzed. Regarding the dependent variable, African American participants had the highest proportion of gambling disorder (55.6%), while white participants had the lowest proportion of gambling disorder (21.2%). The income group with the greatest representation was low income for African Americans (39.8%) and high income for whites (41.5%). Participants in the ‘Other’ race/ethnicity category were more heterogenous for income, with roughly a third in low income, a third between the two middle income categories, and a third in high income. Regarding the remaining independent variables, notable differences included the white group having the highest proportion of alcohol abuse, the ‘Other’ race/ethnicity group being the youngest, and the African American group having the highest proportion of women, those with less than high school education, unemployed, veteran status, divorced/separated/widowed, current smoker, and substance abuse.

**Table 1:** Sample characteristics stratified by race/ethnicity, Prevention and Etiology of Gambling Addiction Study in the United States (PEGASUS), Maryland, 2015

Variable	Total (n = 1346)		African American (n = 630)		White (n = 532)		Other (n = 180)		*p* value^a^
	No.	%^b^	No.	%	No.	%	No.	%	
*Income, annual household*									< 0.0001
Low income									
< $15,000	426	31.7	251	39.8	111	20.9	62	34.4	
Low-middle income									
$15,000 to < $25,000	189	14.0	109	17.3	61	11.5	19	10.6	
High-middle income									
$25,000 to < $50,000	327	24.3	152	24.1	136	25.6	39	21.7	
High income									
$50,000 +	384	28.5	106	16.8	221	41.5	56	31.1	
*Race/ethnicity*	1342	99.7	630	46.8	532	39.5	180	13.4	< 0.0001^c^
*Gambling disorder*^*d*^									< 0.0001
No	827	61.4	279	44.3	418	78.6	127	70.6	
Yes	517	38.4	350	55.6	113	21.2	53	29.4	
Age, years^e^	43	25.6	48.6	21.2	39.1	26.3	28.6	20.9	< 0.0001^f^
*Age, categorical*									< 0.0001
18–24	175	13.0	37	5.9	85	16	53	29.4	
25–34	331	24.6	122	19.4	148	27.8	60	33.3	
35–44	209	15.5	106	16.8	81	15.2	21	11.7	
45–54	314	23.3	187	29.7	102	19.2	23	12.8	
55–64	257	19.1	150	23.8	88	16.5	19	10.6	
65 +	60	4.5	28	4.4	28	5.3	4	2.2	
*Sex*^*g*^									< 0.0001
Male	639	47.5	251	39.8	291	54.7	96	53.3	
Female	700	52	376	59.7	240	45.1	82	45.6	
*Education level*									< 0.0001
< high school graduate	98	7.3	68	10.8	21	4.0	9	5.0	
High school graduate	285	21.2	199	31.6	68	12.8	18	10.0	
> high school graduate	958	71.2	360	57.1	443	83.3	152	84.4	
*Employment status*									< 0.0001
Employed	693	51.5	304	48.3	311	58.5	77	42.8	
Unemployed	331	24.6	211	33.5	88	16.5	31	17.2	
Non-working	307	22.8	108	17.2	130	24.4	68	37.8	
*Veteran*									0.0034
No	1179	87.6	520	82.5	489	91.9	167	92.8	
Yes	95	7.1	59	9.4	27	5.1	9	5.0	
*Marital status*									< 0.0001
Single	764	56.8	342	54.3	294	55.3	127	70.6	
Married	251	18.7	100	15.9	128	24.1	23	12.8	
Divorced, separated, or widowed	327	24.3	185	29.4	110	20.7	30	16.7	
*Homeless*									0.0702 ^h^
No	1313	97.6	608	96.5	523	98.3	179	99.4	
Yes	26	1.9	18	2.9	7	1.3	1	0.6	
*Current smoker*									< 0.0001
No	896	66.6	345	54.8	412	77.4	136	75.6	
Yes	446	33.1	283	44.9	119	22.4	43	23.9	
*Alcohol abuse*^*i*^									0.0014
No	1074	79.8	515	81.8	407	76.5	148	82.2	
Yes	218	16.2	79	12.5	111	20.9	28	15.6	
*Substance abuse*^*j*^									< 0.0001
No	1069	79.4	468	74.3	449	84.4	150	83.3	
Yes	160	11.9	100	15.9	48	9.0	11	6.1	
*Marijuana use in past 30* *days*									0.0808
No	1077	80.0	487	77.3	443	83.3	145	80.6	
Yes	261	19.4	137	21.8	89	16.7	34	18.9	

^a^*p* values from Chi square test for association between variable and racial/ethnic group

^b^Percentages are column percentages (except for ‘Race/ethnicity’ which uses row percentages). Percentages are rounded to the nearest tenth decimal place. Some percentages do not add to 100% due to missingness and/or rounding

^c^*p* value from Chi square test for equal proportions

^d^Gambling disorder defined according to the Diagnostic and Statistical Manual of Mental Disorders, 5th edition (DSM-5) criteria for lifetime gambling disorder as derived from an adapted version of the survey tool Alcohol Use Disorders and Associated Disabilities Interview Schedule-IV (AUDADIS-IV)

^e^Values expressed as median and interquartile range

^f^*p* value from one-way analysis of variance

^g^Three participants who identified as female-to-male transgender were included in female sex group for the analysis

^h^*p* value from Fisher’s exact test

^i^Alcohol abuse defined as those with Alcohol Use Disorder Identification Test score of 8 or greater

^j^Substance abuse defined as those with Drug Abuse Screening Test-20 score of 6 or greater

### Unadjusted Analyses

Unadjusted analysis of the association between income and gambling disorder showed an inverse association, with increasing odds of gambling disorder as income level decreased (Table 2). This association was significant for each level of income evaluated, with the low income group having the greatest odds of gambling disorder (OR 3.79, 95% CI 2.79, 5.15). Addition of interaction terms did not result in significant interaction by sex (global *p* value = 0.77), but did result in a near significant interaction by race/ethnicity (global *p* value = 0.09).

**Table 2:** Association between income level and gambling disorder (all race/ethnicity groups combined), Prevention and Etiology of Gambling Addiction Study in the United States (PEGASUS), Maryland, 2015

	Unadjusted			Adjusted^a^		
	N with/without gambling disorder	OR^b^ (95% CI)	*p* value	N with/without gambling disorder	OR (95% CI)	*p* value
*Income level*						
Low income	221/205	3.79 (2.79, 5.15)	< 0.0001	197/185	2.27 (1.46, 3.53)	0.0003
Low-middle income	79/110	2.53 (1.73, 3.68)	< 0.0001	65/101	1.44 (0.90, 2.29)	0.1278
High-middle income	120/205	2.06 (1.48, 2.86)	< 0.0001	94/183	1.38 (0.93, 2.03)	0.1074
High income	85/299	Referent		77/262	Referent	

*OR* odds ratio, *CI* confidence interval

^a^Adjusted for: age, sex, education, employment, marital status, current smoking, alcohol abuse, substance abuse

^b^Odds ratios from logistic regression

### Adjusted Analyses

After adjusting for potential confounders, the association between low income and gambling disorder attenuated but remained significant (OR 2.27, 95% CI 1.46, 3.53) (Table 2). For the low-middle and high-middle income groups, the association became non-significant. The inverse relationship between income level and odds of gambling disorder persisted, albeit to a lesser degree. When stratifying by race/ethnicity, the global *p* value for the interaction term was not significant (*p* = 0.17), indicating no evidence for effect modification by race/ethnicity (Table 3). This is further supported by the majority of race/ethnicity-income strata having confidence intervals that include 1. The only race/ethnicity-income stratum with a confidence interval that did not include 1 was the ‘Other’ race/ethnicity group of high-middle income. However, the confidence interval was considerably large (1.07, 10.69), and the sample size for this stratum very small (n = 30).

**Table 3:** Adjusted association between income level and gambling disorder stratified by race/ethnicity using a reference group in each stratum, Prevention and Etiology of Gambling Addiction Study in the United States (PEGASUS), Maryland, 2015

	African American		White		Other		*p* value^b^
	N with/without gambling disorder	OR^a^ (95% CI)	N with/without gambling disorder	OR (95% CI)	N with/without gambling disorder	OR (95% CI)	
*Income level*							
Low income	147/76	1.69 (0.92, 3.11)	31/71	1.55 (0.78, 3.06)	19/38	2.65 (0.90, 7.79)	0.1682
Low-middle Income	45/50	0.98 (0.51, 1.88)	15/40	1.19 (0.55, 2.60)	5/11	2.34 (0.57, 9.58)	
High-middle income	64/59	1.42 (0.79, 2.57)	18/106	0.61 (0.31, 1.19)	12/18	3.38 (1.07, 10.69)	
High income	34/55	Referent for African American	36/164	Referent for White	7/43	Referent for other	

*OR* odds ratio, *CI* confidence interval

^a^Odds ratios from logistic regression

^b^Global *p* value for interaction term (race/ethnicity * income) in adjusted analysis (adjusting for: age, sex, education, employment, marital status, current smoking, alcohol abuse, substance abuse)

### Sensitivity Analyses

The sensitivity analyses did not show any difference with regard to effect modification by race/ethnicity. When we repeated the main analysis using a race/ethnicity variable with a greater number of categories, there was still no effect modification by race/ethnicity (global *p* value = 0.80). When employing a past-year diagnosis of gambling disorder as the dependent variable, there was also no effect modification by race/ethnicity (global *p* value = 0.07). The sample sizes for the sensitivity analyses were 1164 for the past-year diagnosis of gambling disorder and 1162 for the alternative race/ethnicity variable analysis.

## Discussion

Although racial/ethnic differences in the prevalence of gambling disorder are well documented, with African Americans having a consistently higher prevalence than whites (Alegría et al. 2009; Gerstein et al. 1999; Welte et al. 2001), underlying reasons for these differences have not been well delineated (Barnes et al. 2017). Our study aimed to explore whether there might be racial/ethnic differences in the influence of income on the odds of gambling disorder among participants in the ongoing PEGASUS cohort study. We found that: (1) There was no evidence that the effect of income varies by race/ethnicity in this cohort, (2) there was a significant association between income and gambling disorder for those in the low income group, even after controlling for multiple confounders.

Our findings are consistent with prior research in several respects. First, regarding racial/ethnic differences in gambling disorder prevalence, several studies have shown that the prevalence of gambling disorder among African Americans is typically double that of whites (Alegría et al. 2009; Gerstein et al. 1999; Welte et al. 2001). Although our study was not a prevalence study (i.e. it involved non-probability sampling), the percentage of African Americans with gambling disorder was more than double that of white participants (55.6% and 21.2%, respectively). Next, regarding the relationship between income and gambling disorder, prior studies suggested an inverse association between income and gambling disorder, with an increasing prevalence of gambling disorder as income decreases (Alegría et al. 2009; Gerstein et al. 1999; Shinogle et al. 2011). Our study confirmed this inverse association, but the adjusted association was only significant for those in the low income group. We suspect this difference is because our study adjusted for confounders whereas prior studies reported unadjusted, descriptive results (Alegría et al. 2009; Gerstein et al. 1999; Shinogle et al. 2011). Finally, regarding studies that evaluate race/ethnicity, income, and gambling disorder, in the 2009 study by Alegría et al., African Americans with “disordered gambling” (which they defined as having three or more DSM-IV criteria) were more likely to be in the low personal income group (< $20,000) compared to whites with “disordered gambling” (Alegría et al. 2009). Our study used different definitions for gambling and income, but similarly showed that African Americans with gambling disorder were more likely to be in the low income group than whites (39.8% and 20.9%, respectively). Lastly, in 2017 Barnes et al. performed an investigation into numerous risk factors for gambling disorder and their interaction with race/ethnicity. They did not find any evidence for interaction by race/ethnicity for the association between socioeconomic status (SES) and “problem gambling” (Barnes et al. 2017). They defined SES using three equally weighted variables: years of education, occupational prestige, and family income. Our results were consistent with their findings, except that we chose to evaluate the effect of income alone rather than as a composite of SES variables.

## Strengths and Limitations

Our study has a couple notable strengths. First, like the analysis by Barnes et al. (2017), rather than simply reporting descriptive statistics, our study takes an analytic approach to understand racial/ethnic differences in the prevalence of gambling disorder. In doing so, we not only see the differences in distribution of income among people with gambling disorder for each race/ethnicity, but we are also able to evaluate this relationship when controlling for potential confounders. Second, the PEGASUS cohort is uniquely suited to studying this research question, since there are a large number of both African American participants and individuals with gambling disorder, thus increasing our ability to detect differences in underlying risk factors.

There are several limitations to our study. First, since participants were recruited by advertisement, selection bias could be present if those who participated are different from the general population with respect to our study question. Second, we were unable to control for psychiatric diagnoses (e.g. depression, anxiety) in this analysis. Since psychiatric comorbidity is closely tied to gambling disorder (Petry et al. 2005), it is possible that inclusion of these variables could have altered our findings. Third, although the PEGASUS sample is a diverse group of individuals, because of small numbers in certain race/ethnicity groups, we chose to create an ‘Other’ race/ethnicity group (n = 180). Although this allowed us to include these individuals in the analysis, it created a heterogenous group for which it can be difficult to draw conclusions. Fourth, it is possible that our results could be influenced by missing data, since we excluded 13.5% of participants due to missing information on variables of interest, and there were significant differences between those included and excluded in our study. Finally, as with any cross-sectional study, we cannot be certain about the assumed temporal relationship between income and gambling disorder, and so reverse causality is possible.

## Implications

Our findings indicate that more research is needed to investigate racial/ethnic differences in gambling disorder in order to inform appropriate prevention and treatment efforts. We suggest that future studies continue to explore differences in underlying risk factors which might help explain racial/ethnic differences in prevalence. Additionally, given the potential for reverse causality with cross-sectional studies, there is a need for future analyses to be made on prospective, longitudinal cohorts (such as the ongoing PEGASUS study).

## Conclusion

In conclusion, despite well-known racial/ethnic differences in gambling disorder prevalence, little is understood about the underlying reasons for such differences (Alegría et al. 2009; Barnes et al. 2017; Gerstein et al. 1999; Welte et al. 2001). Our study examined whether the strength of the association between income and gambling disorder might vary by race/ethnicity. In this analysis, we found no evidence for such variation, indicating that racial/ethnic differences in gambling disorder do not appear to be explained by underlying racial/ethnic differences in income. Instead, for all race/ethnicity groups combined, being in the low income group was associated with significantly increased odds (OR 2.27, 95% CI 1.46, 3.53) of gambling disorder compared to those in the high income group. Further studies exploring other underlying risk factors should be undertaken in order to better understand racial/ethnic differences in gambling disorder prevalence.
